# A short certificateless aggregate signature against coalition attacks

**DOI:** 10.1371/journal.pone.0205453

**Published:** 2018-12-12

**Authors:** Xiaodong Yang, Jinli Wang, Tingchun Ma, Yutong Li, Caifen Wang

**Affiliations:** College of Computer Science and Engineering, Northwest Normal University, Lanzhou, Gansu, China; Victoria University, AUSTRALIA

## Abstract

Certificateless aggregate signature (CLAS) is a crucial cryptosystem. It can not only compress multiple signatures into a short signature, but also ensure the validity of each signature participating in the aggregation by verifying the validity of an resulting aggregate signature. Therefore, a secure and efficient CLAS scheme is very useful for resource-constrained environments because it greatly reduces the overall length of the signature and the verifier’s computational overhead. Cheng et al. presented an efficient CLAS scheme and proved its security in the random oracle model. However, we find that their scheme has security flaws. In this paper, we demonstrate that Cheng et al.’s CLAS scheme is vulnerable to coalition attacks from internal signers. To overcome these attacks, we present an improved CLAS scheme and prove that it is existentially unforgeable under the computational Diffie-Hellman assumption. In addition, our CLAS scheme can not only resist coalition attacks but also generate a very short aggregate signature. The performance analysis results show that our improved CLAS scheme is lower than the related CLAS schemes in terms of communication overhead and computation cost.

## 1 Introduction

Digital signature is one of the major cryptosystems in modern cryptography, which can authenticate the user’s identity, protect the integrity of the data and achieve the non-repudiation of the user’s behavior. Hence, digital signature is one of the important security technologies in open network environments [1–3], which can ensure data security and identity authentication in new network forms and services [4–6]. In a digital signature scheme, the user uses his/her private key to sign the message, while the corresponding public key is used to verify the legality of the signature generated by the user. However, in a traditional public key cryptosystem, the user’s public key is a random string that cannot be associated with the user’s identity. To authenticate the authenticity of the user’s public key, the user’s identity and public key are usually bound by a public key certificate issued by a certificate authority [7–9]. That is, the validity of the user’s public key is determined by the legitimacy of the corresponding certificate. Unfortunately, the storage and management of numerous public key certificates require huge computational and storage overheads when deployed in the real world, which greatly increases the burden of the system.

To eliminate the problem of certificate management in public key cryptosystems, the concept of identity-based cryptography was proposed by Shamir [10]. In an identity-based signature (IBS) scheme, each user selects a unique identity as his/her public key, such as email address, telephone number, etc., and the user’s private key is computed by a trusted private key generator (PKG). Because the user’s public key is a public identity information, it does not need a certificate to verify the validity of the user’s public key. So, the identity-based signature scheme implements the binding of the public key and the entity identity in another way, and greatly simplifies key management. Boneh and Franklin [11] used bilinear pairing to design the first practical identity-based encryption scheme. Later, many efficient IBS schemes have also been presented [12–14]. However, the PKG can generate each user’s private key, which makes the PKG impersonate the user to sign arbitrary message without being discovered. Hence, key escrow is the main security flaw in identity-based cryptography.

Al-Riyami and Paterson [15] introduced the notion of certificateless signature (CLS) to solve the inherent key escrow problem in identity-based cryptosystems. In a CLS scheme, the user’s private key is composed of the secret value and the partial private key. The secret value is selected by the user himself, and the partial private key is generated by a third party called key generation center (KGC). Since the KGC does not know the secret value of the user, PKG cannot obtain the user’s full private key. In this way, the security threats posed by key escrow are resolved. In addition, the user’s public key is derived from the secret value but no longer requires a certificate for authentication. That is, the CLS scheme can basically solve the problems of both the traditional signature scheme and the IBS scheme. Therefore, CLS has become one of the most important research hotspots in public key cryptography. The first CLS scheme was designed by Al-Riyami and Paterson [15], but Huang et al. [16] found that their scheme [15] was insecure under public key replacement attacks. Subsequently, several efforts have been made to construct more secure CLS schemes [17–21].

Certificateless aggregate signature (CLAS) has the advantages of CLS and aggregate signature, which has attracted great attention from researchers. CLAS can compress *n* individual signatures of *n* different messages into a short signature. The verifier only needs to verify the final aggregate signature to determine whether *n* individual signatures involved in the aggregation are valid. As a result, CLAS not only reduces the signature length and the verifier’s computation costs, but also enhances the security of aggregate signature. CLAS is a very useful technique for reducing communication bandwidth and storage overhead, and it is very suitable for environments with limited computing resources and high-efficiency requirements, such as vehicular ad-hoc networks [22–24], wireless sensor networks [25, 26], the Internet of things [27], and so on [28, 29].

In recent years, researchers have proposed many CLAS schemes [30–32]. Yum and Lee [33] presented a CLAS scheme in the random oracle model. However, Hu et al. [34] found that their CLAS scheme [33] was unable to resist public key replacement attacks. Chen et al. [35] presented another CLAS scheme, but was vulnerable to public key replacement attacks and honest-but-curious KGC attacks [36, 37]. Deng et al. [38] constructed an efficient CLAS scheme and demonstrated its security, but Kumar and Sharma [39] showed that this scheme [38] could not provide unforgeability. Zhang et al. [40] also proposed an efficient CLAS scheme and proved its security in the random oracle model. However, Shim [41] pointed out that their CLAS scheme could not resist collusion attacks from internal users and KGC. Xiong et al. [42] designed a CLS scheme with lower signature verification overhead and further constructed a CLAS scheme with higher performance. However, Zhang et al. [43] pointed out that the CLS and CLAS schemes of Xiong et al. [42] could not withstand attacks from a malicious-but-passive KGC. In addition, Cheng et al. [44] and He et al. [45] noted that the CLS and CLAS schemes of Xiong et al. [42] were also insecure against honest-but-curious KGC attacks, respectively. An improved CLAS scheme was proposed by He et al. [45], but Li et al. [46] demonstrated that this modified scheme was still insecure under malicious-but-passive KGC attacks. Cheng et al. [44] also designed an improved version of Xiong et al.’s CLAS scheme, which was proved to be existentially unforgeable in the random oracle model. Unfortunately, we find that Cheng et al.’s CLAS scheme [44] is also insecure against coalition attacks from the collusion of insider signers. That is, the signers generate multiple invalid individual signatures, but the resulting aggregate signature is valid.

To the best of our knowledge, it is still open and challenging to design a secure and efficient CLAS scheme. In this paper, we focus on this issue, providing a new method for addressing it. We first present a coalition attack of insider signers to show the security flaws of Cheng et al.’s CLAS scheme [44]. Such attacks indicate that the validity of an aggregate signature does not guarantee the legitimacy of each individual signatures participating in the aggregation. However, this result has violated the security objectives of the CLAS scheme. To withstand this kind of practical and powerful attack, we further propose an improved CLAS scheme on the basis of Wang et al.’s CLS scheme [47]. In the random oracle model, we prove that the improved CLAS scheme satisfies existential unforgeability under the computational Diffie-Hellman (CDH) assumption. We also demonstrate that our CLAS scheme is secure against coalition attacks. In our CLAS scheme, an aggregate signature is only a group element, so the length of an aggregate signature is independent of the number of signers involved in the aggregation. Additionally, the analysis results indicate that the improved CLAS scheme has lower computational overhead and communication costs.

The remainder of this paper is organized as follows. Section 2 introduces some preliminaries. Section 3 reviews Cheng et al.’s CLAS scheme [44] and gives the corresponding cryptanalysis. Our improved CLAS scheme is presented in Section 4. Section 5 gives the conclusions.

## 2 Preliminaries

The following are some preliminaries related to this paper, including the security notions of CLAS, bilinear paring and complexity assumption.

### 2.1 Formal security model of CLAS

A CLAS scheme mainly includes three entities: KGC, *n* users and an aggregator, as formally defined in the following seven algorithms [32–34].

- **Setup**(λ) → (*pp*, *msk*): Given a security parameter λ, the KGC executes this algorithm to produce the master secret key *msk* and public parameters *pp*.
- **PartialKeyGen**(*pp*, *msk*, *ID*_*i*_) → *psk*_*i*_: Upon input of *pp*, *msk* and a user $U_{i}$’s identity *ID*_*i*_, the KGC executes this algorithm to produce a partial private key *psk*_*i*_ for $U_{i}$.
- **UserKeyGen**(*pp*, *ID*_*i*_) → (*usk*_*i*_, *pk*_*i*_): Upon input of *pp* and *ID*_*i*_, the user $U_{i}$ creates its secret value *usk*_*i*_ and public key *pk*_*i*_. Note that the private key of $U_{i}$ is *sk*_*i*_ = (*usk*_*i*_, *psk*_*i*_).
- **Sign**(*pp*, *sk*_*i*_, *m*_*i*_) → *σ*_*i*_: Upon input of *pp*, *sk*_*i*_ and message *m*_*i*_, the user $U_{i}$ generates an individual signature *σ*_*i*_ on *m*_*i*_.
- **Verify**(*pp*, *ID*_*i*_, *pk*_*i*_, *m*_*i*_, *σ*_*i*_) → {0, 1}: Upon input of *pp*, *ID*_*i*_, *pk*_*i*_ and a signature *σ*_*i*_ on a message *m*_*i*_, this algorithm outputs 1 if *σ*_*i*_ is valid; else, it outputs 0.
- **Aggregate** $(pp,{\left\{m_{i},\sigma_{i}\right\}}_{i=1}^{n})\to \sigma$: Upon input of *n* message/signature pairs (*m*_*i*_, *σ*_*i*_) from *n* users $U_{i}$ (*i* = 1, …, *n*), the aggregator runs this algorithm to generate an aggregate signature *σ* on {*m*_1_, …, *m*_*n*_}.
- **AggVerify** $\left(pp,\sigma ,{\left\{ID_{i},pk_{i},m_{i}\right\}}_{i=1}^{n}\right)\to \left\{0,1\right\}$: Given *pp* and an aggregate signature *σ* of *n* messages {*m*_1_, …, *m*_*n*_} on {*ID*_1_, …, *ID*_*n*_} and {*pk*_1_, …, *pk*_*n*_}, this algorithm outputs 1 if *σ* is valid; else, it outputs 0.

The security model of a CLAS scheme mainly considers two types of attackers [42, 44, 48], named Type I adversary $A_{1}$ and Type II adversary $A_{2}$.

- $A_{1}$ represents a dishonest user (i.e., an external attacker). $A_{1}$ can know the secret value of each user and replace any user’s public key, but does not know the master secret key *msk*.
- $A_{2}$ represents a malicious KGC. A honest-but-curious KGC knows *msk* and generates the user’s private key, but is unable to perform public key replacement. In addition to having the ability of a honest-but-curious KGC, a malicious-but-passive KGC is also allowed to embed trapdoors in *msk* and public parameters *pp* during the system initialization phase.

The following uses two security games between an attacker $A\in \{A_{1},A_{2}\}$ and a challenger C to define the unforgeability of a CLAS scheme.

**Game 1**: The first game is played by C and $A_{1}$.

- *Initialization*: C sends public parameters *pp* generated by the algorithm **Setup**(λ) to $A_{1}$, and then secretly stores the master secret key *msk*.
- *Partial-private-key-query*: If $A_{1}$ asks for a partial private key of an identity *ID*_*i*_, then C executes the algorithm **PartialKeyGen**(*pp*, *msk*, *ID*_*i*_) to produce *psk*_*i*_ and returns it to $A_{1}$.
- *Public-key-query*: If $A_{1}$ requests a public key of *ID*_*i*_, then C performs the algorithm **UserKeyGen**(*pp*, *ID*_*i*_) to produce *pk*_*i*_ and returns it to $A_{1}$.
- *Secret-value-query*: If C receives an identity *ID*_*i*_ sent by $A_{1}$, then C responds to $A_{1}$ with a secret value *usk*_*i*_ output by the algorithm **UserKeyGen**(*pp*, *ID*_*i*_).
- *Public-key-replacement-query*: If C receives *ID*_*i*_ and $pk_{i}^{{}^{\prime }}$ sent by $A_{1}$, then the public key of *ID*_*i*_ is replaced by $pk_{i}^{{}^{\prime }}$.
- *Sign-query*: When $A_{1}$ asks for a signature of a message *m*_*i*_ and an identity *ID*_*i*_, C runs the algorithm **Sign**(*pp*, *sk*_*i*_, *m*_*i*_) to produce an individual signature *σ*_*i*_ of *m*_*i*_ and sends it to $A_{1}$.
- *Forgery*: $A_{1}$ finally produces a forgery $\left({\left\{m_{i}^{*},ID_{i}^{*},pk_{i}^{*},\sigma_{i}^{*}\right\}}_{i=1}^{n},\sigma^{*}\right)$, where at least one of *n* identities $\left\{ID_{1}^{*},...,ID_{n}^{*}\right\}$ is the identity ID* of the target user. Assumed that the *t*-th signer’s identity $ID_{t}^{*}=ID^{*}$ and that $\sigma_{t}^{*}$ is an individual signature of $\left(ID_{t}^{*},pk_{t}^{*}\right)$ on message $m_{t}^{*}$, where *t* ∈ {1, …, *n*}. If the following three conditions are satisfied, then $A_{1}$ wins the game.
  1. *Partial-private-key-query* has never received an inquiry about $ID_{t}^{*}$.
  2. *Sign-query* has never received an inquiry about $\left(ID_{t}^{*},m_{t}^{*}\right)$.
  3. σ* is a valid aggregate signature of ${\left\{ID_{i}^{*},pk_{i}^{*}\right\}}_{i=1}^{n}$ on messages ${\left\{m_{i}^{*}\right\}}_{i=1}^{n}$.

**Game 2**: The second game is played by C and $A_{2}$. We consider that the second type of attacker is a malicious-but-passive KGC, which is a strengthened security model.

- *Initialization*: $A_{2}$ sends the master secret key *msk* and public parameters *pp* generated by the algorithm **Setup**(λ) to C.
- *Queries*: In addition to *Partial-private-key-query* and *Public-key-replacement-query*, $A_{2}$ may initiate other queries defined in Game 1.
- *Forgery*: $A_{2}$ finally produces a forgery $\left({\left\{m_{i}^{*},ID_{i}^{*},pk_{i}^{*},\sigma_{i}^{*}\right\}}_{i=1}^{n},\sigma^{*}\right)$. Assume that the target user’s identity $ID^{*}=ID_{t}^{*}$ and that $\sigma_{t}^{*}$ is an individual signature of (ID*, pk*) on message $m_{t}^{*}$, where *t* ∈ {1, …, *n*}. If the following three conditions are satisfied, then $A_{2}$ wins the game.
  1. *Secret-value-query* has never received an inquiry about $ID_{t}^{*}$.
  2. *Sign-query* has never received an inquiry about $\left(ID_{t}^{*},m_{t}^{*}\right)$.
  3. σ* is a valid aggregate signature of ${\left\{ID_{i}^{*},pk_{i}^{*}\right\}}_{i=1}^{n}$ on messages ${\left\{m_{i}^{*}\right\}}_{i=1}^{n}$.

**Definition 1**. If there is no polynomial-time attacker $A\in \{A_{1},A_{2}\}$ who can win in the above two games with a non-negligible probability, then a CLAS scheme is said to be existentially unforgeable under adaptive chosen-message attacks [42, 44, 48].

### 2.2 Bilinear pairing and complexity assumption

Assume that *p* is a prime number, *G*_1_ and *G*_2_ are two cyclic groups with order *p*, and *g* is a generator of *G*_1_. If a map *e*: *G*_1_ × *G*_1_ → *G*_2_ satisfies the following conditions, then *e* is called a bilinear pairing [43]:

- *Bilinearity*: For all *γ*_1_, *γ*_2_ ∈ *Z*_*p*_, $e\left(g^{\gamma_{1}},g^{\gamma_{2}}\right)=e{\left(g,g\right)}^{\gamma_{1}\gamma_{2}}=e\left(g^{\gamma_{2}},g^{\gamma_{1}}\right)$.
- *Non-degeneracy*:*e*(*g*, *g*) is not an identity element $1_{G_{2}}$ in *G*_2_. That is, $e\left(g,g\right)\neq 1_{G_{2}}$.
- *Computability*: For any *γ*_1_, *γ*_2_ ∈ *Z*_*p*_, an algorithm for efficiently calculating $e(g^{\gamma_{1}},g^{\gamma_{2}})$ exists.

Given three elements $\left(g,g^{a},g^{b}\right)\in G_{1}^{3}$, the CDH problem is to calculate *g*^*ab*^ ∈ *G*_1_, where the unknown values *a* and *b* are randomly chosen from $Z_{p}^{*}$.

**Definition 2 (CDH assumption)**. If the probability of any polynomial-time attacker to solve the CDH problem is negligible, then the CDH problem is called intractable [47, 49].

## 3 Cryptanalysis of Cheng et al.’s CLAS scheme

### 3.1 Review of Cheng et al.’s CLAS scheme

To overcome the security flaws in Xiong et al.’s CLAS scheme [42], Cheng et al. [44] presented an improved CLAS scheme that is described as follows.

- **Setup**: Given a security parameter λ, the KGC selects two multiplicative cyclic groups *G*_1_ and *G*_2_ with prime order *p*, a bit string *Q* of length *l*, a generator *g* in *G*_1_ and a bilinear pairing *e*: *G*_1_ × *G*_1_ → *G*_2_. Then, the KGC randomly selects $s\in Z_{p}^{*}$ and computes *P*_*pub*_ = *g*^*s*^. The KGC also picks three hash functions *H*_0_: {0, 1}^*l*^ → *G*_1_, *H*_1_: {0, 1}* → *G*_1_ and $H_{2}:{\left\{0,1\right\}}^{*}\to Z_{p}^{*}$. Finally, the KGC secretly stores the master secret key *msk* = *s* and publishes public parameters *pp* = {*G*_1_, *G*_2_, *p*, *g*, *e*, *Q*, *P*_*pub*_, *H*_0_, *H*_1_, *H*_2_}.
- **PartialKeyGen**: After receiving an identity *ID*_*i*_ submitted by a user $U_{i}$, the KGC computes *psk*_*i*_ = *H*_1_(*ID*_*i*_)^*s*^ and sends *ID*_*i*_’s partial private key *psk*_*i*_ to $U_{i}$ through a secure channel.
- **UserKeyGen**: The user $U_{i}$ with identity *ID*_*i*_ randomly selects $x_{i}\in Z_{p}^{*}$ and computes public key $pk_{i}=g^{x_{i}}$. Then, $U_{i}$ sets its secret value *usk*_*i*_ = *x*_*i*_ and private key *sk*_*i*_ = (*usk*_*i*_, *psk*_*i*_) = (*x*_*i*_, *H*_1_(*ID*_*i*_)^*s*^).
- **Sign**: For a message *m*_*i*_, the signer $U_{i}$ selects a random value $r_{i}\in Z_{p}^{*}$ and computes $R_{i}=g^{r_{i}}$, *h*_*i*_ = *H*_2_(*m*_*i*_, *ID*_*i*_, *pk*_*i*_, *R*_*i*_) and $V_{i}=psk_{i}\cdot {\left(P_{pub}\right)}^{h_{i}r_{i}}\cdot H_{0}{\left(Q\right)}^{h_{i}x_{i}+r_{i}}$. Then, $U_{i}$ outputs *σ*_*i*_ = (*R*_*i*_, *V*_*i*_) as an individual signature of *m*_*i*_.
- **Verify**: Given *ID*_*i*_, *pk*_*i*_ and an individual signature *σ*_*i*_ = (*R*_*i*_, *V*_*i*_) on message *m*_*i*_, a verifier computes *H*_0_(*Q*), *H*_1_(*ID*_*i*_) and *h*_*i*_ = *H*_2_(*m*_*i*_, *ID*_*i*_, *pk*_*i*_, *R*_*i*_) and then verifies the following equation:
  $$e\left(V_{i},g\right)=e\left(R_{i}^{h_{i}}H_{1}\left(ID_{i}\right),P_{pub}\right)e\left(pk_{i}^{h_{i}}R_{i},H_{0}\left(Q\right)\right).$$
  If this equation holds, output 1; otherwise, output 0.
- **Aggregate**: After receiving the message-signature pair (*m*_*i*_, *σ*_*i*_ = (*R*_*i*_, *V*_*i*_)) from user $U_{i}$ for *i* = 1, …, *n*, the aggregator calculates $V=\prod_{i=1}^{n}V_{i}$ and outputs an aggregate signature *σ* = (*R*_1_, …, *R*_*n*_, *V*).
- **AggVerify**: Given *n* identities {*ID*_1_, …, *ID*_*n*_}, *n* public keys {*pk*_1_, …, *pk*_*n*_}, *n* messages {*m*_1_, …, *m*_*n*_} and an aggregate signature *σ* = (*R*_1_, …, *R*_*n*_, *V*), a verifier first computes *H*_0_(*Q*), *H*_1_(*ID*_*i*_) and *h*_*i*_ = *H*_2_(*m*_*i*_, *ID*_*i*_, *pk*_*i*_, *R*_*i*_) for *i* = 1, …, *n*. Then, the verifier verifies the following equation:
  $$e\left(V,g\right)=e\left(\prod_{i=1}^{n}R_{i}^{h_{i}}H_{1}\left(ID_{i}\right),P_{pub}\right)e\left(\prod_{i=1}^{n}pk_{i}^{h_{i}}R_{i},H_{0}\left(Q\right)\right).$$
  If this equation holds, output 1; otherwise, output 0.

### 3.2 Attack on Cheng et al.’s CLAS scheme

In the random oracle model, Cheng et al.’s CLAS scheme [44] was proved to satisfy the security requirement of existential unforgeability. Unfortunately, in this subsection, we demonstrate that Cheng et al.’s CLAS scheme [44] cannot resist coalition attacks from internal signers. Without loss of generality, suppose that {*ID*_1_, *pk*_1_} and {*ID*_2_, *pk*_2_} are the identity/public key pairs of the signers $\left\{U_{1},U_{2}\right\}$, respectively. As a result, we show that $U_{1}$ cooperates with $U_{2}$ to generate invalid individual signature *σ*_*i*_ on message *m*_*i*_ for *i* = 1, 2, whereas the resulting aggregate signature *σ* on {*m*_1_, *m*_2_} is valid.

1. $U_{1}$ randomly selects $r_{1}\in Z_{p}^{*}$ and computes $R_{1}^{*}=g^{r_{1}}$ and $h_{1}=H_{2}\left(m_{1},ID_{1},pk_{1},R_{1}^{*}\right)$. Then, $U_{1}$ sends ${\left(P_{pub}\right)}^{h_{1}r_{1}}$ to $U_{2}$.
2. $U_{2}$ randomly selects $r_{2}\in Z_{p}^{*}$ and computes $R_{2}^{*}=g^{r_{2}}$ and $h_{2}=H_{2}\left(m_{2},ID_{2},pk_{2},R_{2}^{*}\right)$. Then, $U_{2}$ sends ${\left(P_{pub}\right)}^{h_{2}r_{2}}$ to $U_{1}$.
3. $U_{1}$ computes $V_{1}^{*}=psk_{1}\cdot {\left(P_{pub}\right)}^{h_{2}r_{2}}\cdot H_{0}{\left(Q\right)}^{x_{1}h_{1}+r_{1}}$ and sets $\sigma_{1}^{*}=\left(R_{1}^{*},V_{1}^{*}\right)$ as a signature on *m*_1_.
4. $U_{2}$ computes $V_{2}^{*}=psk_{2}\cdot {\left(P_{pub}\right)}^{h_{1}r_{1}}\cdot H_{0}{\left(Q\right)}^{x_{2}h_{2}+r_{2}}$ and sets $\sigma_{2}^{*}=\left(R_{2}^{*},V_{2}^{*}\right)$ as a signature on *m*_2_.
5. $U_{1}$ cooperates with $U_{2}$ to produce an aggregate signature $\sigma *=\left(R_{1}^{*},R_{2}^{*},V*\right)$ on messages {*m*_1_, *m*_2_}, where $V*=V_{1}^{*}\cdot V_{2}^{*}$.

Clearly, $\left\{\sigma_{1}^{*},\sigma_{2}^{*}\right\}$ are invalid individual signatures of $\left\{U_{1},U_{2}\right\}$ on {*m*_1_, *m*_2_}, respectively. However, the forged aggregate signature $\sigma *=\left(R_{1}^{*},R_{2}^{*},V*\right)$ is valid since

$$\begin{array}{cc} & e\left(V^{*},g\right)=e\left(V_{1}^{*}V_{2}^{*},g\right)\\ = & e(psk_{1}{\left(P_{pub}\right)}^{h_{2}r_{2}}H_{0}{\left(Q\right)}^{x_{1}h_{1}+r_{1}}psk_{2}\cdot {\left(P_{pub}\right)}^{h_{1}r_{1}}H_{0}{\left(Q\right)}^{x_{2}h_{2}+r_{2}},g)\\ = & e(psk_{1}{\left(P_{pub}\right)}^{h_{1}r_{1}}H_{0}{\left(Q\right)}^{x_{1}h_{1}+r_{1}}psk_{2}\cdot {\left(P_{pub}\right)}^{h_{2}r_{2}}H_{0}{\left(Q\right)}^{x_{2}h_{2}+r_{2}},g)\\ = & e\left(\prod_{i=1}^{2}R_{i}^{h_{i}}H_{1}\left(ID_{i}\right),P_{pub}\right)e\left(\prod_{i=1}^{2}pk_{i}^{h_{i}}R_{i},H_{0}\left(Q\right)\right). \end{array}$$

Therefore, dishonest signers can jointly forge a valid aggregate signature for arbitrary messages. This shows that even if some individual signatures participating in the aggregation are invalid, the resulting aggregate signature is also valid. That is, the validity of every individual signature involved in the aggregation cannot be guaranteed by the legitimacy of the corresponding aggregate signature. So, Cheng et al.’s CLAS scheme [44] is insecure under coalition attacks.

## 4 Our improved CLAS scheme

In Cheng et al.’s CLAS scheme [44], dishonest signers can use some invalid individual signatures to jointly generate a valid aggregate signature. However, the main reason for the success of such attacks is due to the fact that signers can exchange their individual signature values with each other during the generation of an aggregate signature.

### 4.1 Construction

In this subsection, we design an enhanced CLAS scheme for preventing the above coalition attack. The following is the description of our CLAS schem.

- **Setup**: The KGC selects two groups *G*_1_ and *G*_2_, which have the same prime order *p*. The KGC randomly selects a generator *g* in *G*_1_ and a bilinear pairing *e*: *G*_1_ × *G*_1_ → *G*_2_. The KGC picks hash functions *H*: {0, 1}* → {0, 1}^*l*^ and *H*_1_, *H*_3_: {0,1}* → *G*_1_. The KCG also chooses a random value $s\in Z_{p}^{*}$ and calculates *P*_*pub*_ = *g*^*s*^. Finally, the KGC publishes public parameters *pp* = {*G*_1_, *G*_2_, *p*, *g*, *e*, *P*_*pub*_, *H*, *H*_1_, *H*_3_} and secretly stores the master secret key *msk* = *s*.
- The algorithms **PartialKeyGen** and **UserKeyGen** are the same as those in the CLAS scheme of Cheng et al.
- **Sign**: For a message *m*_*i*_, the user $U_{i}$ uses its secret value *usk*_*i*_ = *x*_*i*_ and partial private key *psk*_*i*_ = *H*_1_(*ID*_*i*_)^*s*^ to calculate *V*_*i*_ = *H*_3_(*m*_*i*_, *ID*_*i*_, *pk*_*i*_) and
  $$\sigma_{i}=V_{i}^{x_{i}}\cdot psk_{i}.$$
  Then, $U_{i}$ outputs *σ*_*i*_ as an individual signature of *m*_*i*_.
- **Verify**: Given *ID*_*i*_, *pk*_*i*_ and an individual signature *σ*_*i*_ on message *m*_*i*_, a verifier computes *H*_1_(*ID*_*i*_) and *V*_*i*_ = *H*_3_(*m*_*i*_, *ID*_*i*_, *pk*_*i*_), and then checks the following equation:
  $$e(\sigma_{i},g)=e(V_{i},pk_{i})e(H_{1}(ID_{i}),P_{pub}).$$
  If it holds, accept *σ*_*i*_ and output 1; else, reject *σ*_*i*_ and output 0.
- **Aggregate**: Given the message-signature pair (*m*_*i*_, *σ*_*i*_) from user $U_{i}$ for *i* = 1, …, *n*, the aggregator calculates
  $$\sigma =H(e\left(\sigma_{1},g\right),\ldots ,e\left(\sigma_{n},g\right))$$
  and outputs an aggregate signature *σ* on {*m*_1_, …, *m*_*n*_}.
- **AggVerify**: Given *n* identities {*ID*_1_, …, *ID*_*n*_}, *n* public keys {*pk*_1_, …, *pk*_*n*_}, *n* messages {*m*_1_, …, *m*_*n*_} and an aggregate signature *σ*, a verifier computes *H*_1_(*ID*_*i*_) and *V*_*i*_ = *H*_3_(*m*_*i*_, *ID*_*i*_, *pk*_*i*_) for *i* = 1, …, *n*. Then, the verifier checks the following equation:
  $$\begin{array}{c} \sigma =H(e\left(V_{1},pk_{1}\right)e\left(H_{1}\left(ID_{1}\right),P_{pub}\right),\ldots ,e\left(V_{n},pk_{n}\right)e\left(H_{1}\left(ID_{n}\right),P_{pub}\right)). \end{array}$$
  If the above equation holds, accept *σ* as a valid aggregate signature and output 1; else, reject it and output 0.

### 4.2 Correctness

The correctness of verifying an individual signature *σ*_*i*_ on message *m*_*i*_ is given below:

$$\begin{array}{ll} e\left(\sigma_{i},g\right) & =e\left(V_{i}^{x_{i}}\cdot psk_{i},g\right)\\ & =e\left(V_{i}^{x_{i}},g\right)e\left(psk_{i},g\right)\\ & =e\left(V_{i},g^{x_{i}}\right)e\left(H_{1}{\left(ID_{i}\right)}^{s},g\right)\\ & =e\left(V_{i},pk_{i}\right)e\left(H_{1}\left(ID_{i}\right),P_{pub}\right). \end{array}$$

The correctness of verifying an aggregate signature *σ* on messages {*m*_1_, …, *m*_*n*_} is given below:

$$\begin{array}{ll} \sigma & =H\left(e\left(\sigma_{1},g\right),\ldots ,e\left(\sigma_{n},g\right)\right)\\ & =H\left(e\left(V_{1},pk_{1}\right)e\left(H_{1}\left(ID_{1}\right),P_{pub}\right),\ldots ,e\left(V_{n},pk_{n}\right)e\left(H_{1}\left(ID_{n}\right),P_{pub}\right)\right). \end{array}$$

From the above analysis, we can see that our CLAS scheme is correct.

### 4.3 Security analysis

We prove that the improved CLAS scheme is existentially unforgeable under Type I and Type II adversaries. Also, we demonstrate that our CLAS scheme can withstand coalition attacks.

**Theorem 1**. If the security of our CLAS scheme is broken by a polynomial-time Type I adversary $A_{1}$, then the CDH problem can be solved.

**Proof**. Suppose that $A_{1}$ forges a valid aggregate signature with probability *ε*_1_ after making at most *q*_*i*_ random oracle queries to *H*_*i*_(*i* = 1, 3), *q*_*psk*_ partial private key queries, *q*_*pk*_ public key queries, *q*_*rep*_ public key replacement queries, *q*_*usk*_ secret value queries and *q*_*s*_ signing queries, then we can construct an algorithm C to solve the CDH problem. After receiving a random CDH instance $\left(g,g^{a},g^{b}\right)\in G_{1}^{3}$, C will utilize $A_{1}$’s forgery to calculate the CDH value *g*^*ab*^.

- *Initialization*: C sets *P*_*pub*_ = *g*^*a*^ and runs the algorithm **Setup**(λ) to produce other parameters. Then, C sends public parameters *pp* to $A_{1}$. Note that the master secret key *msk* = *a* is unknown to C. Moreover, C controls two random oracles to simulate hash functions *H*_1_ and *H*_3_. To simplify the description, it is assumed that $A_{1}$ has performed related hash queries before making other inquiries. To avoid collisions and consistently respond to all kinds of queries initiated by $A_{1}$, C maintains a list $L=(ID_{i},psk_{i},usk_{i},pk_{i})$ and two other lists $\left(L_{1},L_{3}\right)$ that are all initially empty. C picks a random integer *d* ∈ [1, *q*_1_] and responds to all queries initiated by $A_{1}$ in the following way.
- *H*_1_ *queries*: Upon receiving the *i*-th *H*_1_ inquiry about an identity *ID*_*i*_, C first checks whether an entry for *ID*_*i*_ exists in $L_{1}$. If yes, C sends the corresponding value *H*_1_(*ID*_*i*_) to $A_{1}$. Otherwise, C randomly selects $y_{i}\in Z_{p}^{*}$ and sets
  $$H_{1}\left(ID_{i}\right)=\{\begin{array}{c} \begin{array}{cc} g^{y_{i}} & i\neq d \end{array}\\ \begin{array}{cc} {\left(g^{b}\right)}^{y_{i}} & i=d \end{array} \end{array}.$$
  If *i* ≠ *d*, C adds (*ID*_*i*_, *y*_*i*_, *H*_1_(*ID*_*i*_)) to $L_{1}$; otherwise, C sets the target user’s identity ID* = *ID*_*d*_ and y* = *y*_*d*_, and then adds (ID*, y*, *H*_1_(ID*)) to $L_{1}$. Finally, C sends *H*_1_(*ID*_*i*_) to $A_{1}$.
- *H*_3_ *queries*: $A_{1}$ issues an *H*_3_ query for (*m*_*i*_, *ID*_*i*_, *pk*_*i*_). If it is found in $L_{3}$, then C sends the corresponding value *V*_*i*_ to $A_{1}$. Otherwise, C randomly selects $z_{i}\in Z_{p}^{*}$ and sets $V_{i}=H_{3}\left(m_{i},ID_{i},pk_{i}\right)=g^{z_{i}}$. Next, C sends *V*_*i*_ to $A_{1}$ and adds (*m*_*i*_, *ID*_*i*_, *pk*_*i*_, *z*_*i*_, *V*_*i*_) to $L_{3}$.
- *Partial-private-key-query*: After receiving an identity *ID*_*i*_ submitted by $A_{1}$, C considers the following two cases.
  - If *ID*_*i*_ = ID*, C terminates the game.
  - If *ID*_*i*_ ≠ ID*, C checks whether L contains an entry for *ID*_*i*_.
    1. If it exists, C sends *psk*_*i*_ to $A_{1}$.
    2. Else, C finds the tuple (*ID*_*i*_, *y*_*i*_, *H*_1_(*ID*_*i*_)) in $L_{1}$ and returns a partial private key $psk_{i}={\left(P_{pub}\right)}^{y_{i}}$ to $A_{1}$. Then, C adds (*ID*_*i*_, *psk*_*i*_, ⊥, ⊥) to L.
- *Public-key-query*: On receiving an identity *ID*_*i*_, C checks if an entry for *ID*_*i*_ exists in L, where *pk*_*i*_ ≠ ⊥. If yes, C sends *pk*_*i*_ to $A_{1}$. Otherwise, C randomly selects $x_{i}\in Z_{p}^{*}$ and sets *usk*_*i*_ = *x*_*i*_ and $pk_{i}=g^{x_{i}}$. If *ID*_*i*_ = ID*, C sets the target user’s public key pk* = *pk*_*i*_ and secret value usk* = *usk*_*i*_. Then, C adds the corresponding tuple to L and returns a public key *pk*_*i*_ to $A_{1}$.
- *Secret-value-query*: If $A_{1}$ issues a query for an identity *ID*_*i*_, C first initiates a public key query about *ID*_*i*_ and then returns the corresponding secret value *usk*_*i*_ to $A_{1}$.
- *Public-key-replacement-query*: If $A_{1}$ submits an identity *ID*_*i*_ and a public key $pk_{i}^{{}^{\prime }}$, C first checks whether there is an entry for *ID*_*i*_ in L. If yes, C replaces *ID*_*i*_’s public key with $pk_{i}^{{}^{\prime }}$; otherwise, C sets *ID*_*i*_’s public key to $pk_{i}^{{}^{\prime }}$ and then adds a new tuple (*ID*_*i*_, ⊥, ⊥, *pk*_*i*_) in L.
- *Sign-query*: $A_{1}$ asks a signature query on its selected identity *ID*_*i*_ and message *m*_*i*_. If *ID*_*i*_ = ID*, C terminates the game. Else, C searches the lists L and $L_{3}$ to find the corresponding tuples (*psk*_*i*_, *usk*_*i*_, *pk*_*i*_) and *V*_*i*_ = *H*_3_(*m*_*i*_, *ID*_*i*_, *pk*_*i*_), respectively. Then, C returns $\sigma_{i}=V_{i}^{usk_{i}}\cdot psk_{i}$ to $A_{1}$ as a signature on *m*_*i*_.
- *Forgery*: $A_{1}$ eventually produces a valid forgery $\left({\left\{m_{i}^{*},ID_{i}^{*},pk_{i}^{*},\sigma_{i}^{*}\right\}}_{i=1}^{n},\sigma^{*}\right)$. Suppose that the individual signature $\sigma_{t}^{*}$ is not returned by *Sign-query* on the target identity $ID_{t}^{*}$ and message $m_{t}^{*}$, where *t* ∈ {1, …, *n*}. If $ID^{*}\neq ID_{t}^{*}$ or $V^{*}=H_{3}\left(m_{t}^{*},ID^{*},pk^{*}\right)$ is not found in $L_{3}$, C aborts. Else, C finds (usk* = x*, pk*) corresponding to $ID_{t}^{*}$ in L. Then, C obtains the tuples (ID*, y*, *H*_1_(ID*)) and $\left(m_{t}^{*},ID^{*},pk^{*},z^{*},V^{*}\right)$ from $L_{1}$ and $L_{3}$, respectively. Since $\sigma_{t}^{*}$ is valid, the following equation must hold:
  $$\begin{array}{ll} \sigma_{t}^{*} & ={\left(V*\right)}^{x*}H_{1}{\left(ID*\right)}^{a}={\left(V*\right)}^{x*}{\left(g^{by*}\right)}^{a}\\ & ={\left(V*\right)}^{x*}{\left(g^{ab}\right)}^{y*}. \end{array}$$
  From the above equation, C calculates *g*^*ab*^ as
  $$g^{ab}={\left(\frac{\sigma_{t}^{*}}{{\left(V*\right)}^{x*}}\right)}^{{\left(y*\right)}^{-1}}.$$
  Hence, C solves the CDH problem.

Next, we analyze the successful probability that C can solve the CDH problem instance. If the following events occur, C will not quit during the entire simulation game.

- During the simulation of *Partial-private-key-query*, each identity *ID*_*i*_ queried by $A_{1}$ is not equal to ID*. The probability is at least ${\left(1-\frac{1}{q_{1}}\right)}^{q_{psk}}$.
- During the simulation of *Sign-query*, each identity *ID*_*i*_ queried by $A_{1}$ is not equal to ID*. The probability is at least ${\left(1-\frac{1}{q_{1}}\right)}^{q_{s}}$.
- In the forgery phase, at least one of *n* identities $\left\{ID_{1}^{*},\ldots ,ID_{n}^{*}\right\}$ is the target user’s identity ID*. The probability of $ID^{*}=ID_{t}^{*}$, where *t* ∈ {1, …, *n*}, is at least $1-{\left(1-\frac{1}{q_{1}}\right)}^{n}$.
- V* can be found in $L_{3}$. The probability is at least $\left(1-\frac{q_{3}}{p}\right)$.

Hence, the overall success probability of C solving the CDH problem is

$$\begin{array}{ll} \varepsilon_{1}^{{}^{\prime }} & {\geq (1-\frac{1}{q_{1}})}^{q_{psk}}{\left(1-\frac{1}{q_{1}}\right)}^{q_{s}}\left(1-{\left(1-\frac{1}{q_{1}}\right)}^{n}\right)\left(1-\frac{q_{3}}{p}\right)\varepsilon_{1}\\ & ={\left(1-\frac{1}{q_{1}}\right)}^{q_{psk}+q_{s}}\left(1-{\left(1-\frac{1}{q_{1}}\right)}^{n}\right)\left(1-\frac{q_{3}}{p}\right)\varepsilon_{1}. \end{array}$$

**Theorem 2**. If the security of our CLAS scheme is broken by a polynomial-time Type II adversary $A_{2}$, then the CDH problem can be solved.

**Proof**. Let $A_{2}$ be a malicious-but-passive KGC. Suppose that $A_{2}$ forges a valid aggregate signature with probability *ε*_2_ after making at most *q*_*i*_ random oracle queries to *H*_*i*_(*i* = 1, 3), *q*_*pk*_ public key queries, *q*_*s*_ signing queries and *q*_*usk*_ secret value queries, then there exists a solver C that invokes $A_{2}$ to violate the CDH assumption. Given a CDH instance $\left(g,g^{a},g^{b}\right)\in G_{1}^{3}$, the goal of C is to calculate *g*^*ab*^ by interacting with $A_{2}$.

- *Initialization*: $A_{2}$ picks a random value $s\in Z_{p}^{*}$ as the master secret key *msk* and calculates *P*_*pub*_ = *g*^*s*^. Then, $A_{2}$ runs **Setup**(λ) to produce other parameters and sends (*pp*, *msk*) to C. After that, C picks a random integer *d* ∈ [1, *q*_3_] and maintains three initially empty lists L, $L_{1}$ and $L_{3}$.
- *H*_1_ *queries*: Upon receiving an *H*_1_ query on an identity *ID*_*i*_, C returns the corresponding value *H*_1_(*ID*_*i*_) to $A_{2}$ if $L_{1}$ contains an entry for *ID*_*i*_. Otherwise, C selects $y_{i}\in Z_{p}^{*}$ at random and sets $H_{1}\left(ID_{i}\right)=g^{y_{i}}$. Then, C sends $g^{y_{i}}$ to $A_{2}$ and adds (*ID*_*i*_, *y*_*i*_, *H*_1_(*ID*_*i*_)) to $L_{1}$.
- *H*_3_ *queries*: Upon receiving the *i*-th *H*_3_ query on (*m*_*i*_, *ID*_*i*_, *pk*_*i*_), C sends the corresponding *V*_*i*_ to $A_{2}$ if an entry for (*m*_*i*_, *ID*_*i*_, *pk*_*i*_) exists in $L_{3}$. Otherwise, C selects a random value $z_{i}\in Z_{p}^{*}$ and sets
  $$V_{i}=H_{3}\left(m_{i},ID_{i},pk_{i}\right)=\{\begin{array}{c} \begin{array}{cc} g^{z_{i}} & i\neq d \end{array}\\ \begin{array}{cc} {\left(g^{b}\right)}^{z_{i}} & i=d \end{array} \end{array}.$$
  If *i* ≠ *d*, C adds (*m*_*i*_, *ID*_*i*_, *pk*_*i*_, *z*_*i*_, *V*_*i*_) to $L_{3}$; otherwise, C sets the target user’s identity ID* = *ID*_*i*_ and pk* = *pk*_*i*_ and then adds (*m*_*i*_, ID*, pk*, *z*_*i*_, *V*_*i*_) to $L_{3}$. Finally, C sends *V*_*i*_ to $A_{2}$.
- *Public-key-query*: $A_{2}$ asks a public key query for *ID*_*i*_ and if it is found in L, then C returns *pk*_*i*_ to $A_{2}$. Otherwise, C selects a random value $x_{i}\in Z_{p}^{*}$ and sets
  $$pk_{i}=\{\begin{array}{c} \begin{array}{cc} g^{x_{i}} & ID_{i}\neq ID* \end{array}\\ \begin{array}{cc} g^{a} & ID_{i}=ID* \end{array} \end{array}.$$
  If *ID*_*i*_ ≠ ID*, C sets *usk*_*i*_ = *x*_*i*_ and adds (*ID*_*i*_, *x*_*i*_, *pk*_*i*_) to L. Otherwise, C sets the public key pk* = *pk*_*i*_ of the target user, and adds (ID*, ⊥, pk*) to L. Finally, C returns *pk*_*i*_ to $A_{2}$. Note that the target user’s secret value usk* = *a*, while *a* is unknown to C.
- *Secret-value-query*: On receiving the query on *ID*_*i*_, C aborts if *ID*_*i*_ = ID*; otherwise, C first initiates a public key query about *ID*_*i*_ and then returns the corresponding secret value *usk*_*i*_ to $A_{2}$.
- *Sign-query*: Upon receiving an identity *ID*_*i*_ and a message *m*_*i*_, C proceeds as follows:
  - If *ID*_*i*_ = ID*, C aborts.
  - Else, C searches the lists L and $L_{1}$ to find (*ID*_*i*_, *x*_*i*_, *pk*_*i*_) and (*ID*_*i*_, *y*_*i*_, *H*_1_(*ID*_*i*_)), respectively. Next, C makes an *H*_3_ query on (*m*_*i*_, *ID*_*i*_, *pk*_*i*_) to obtain *V*_*i*_ = *H*_3_(*m*_*i*_, *ID*_*i*_, *pk*_*i*_). Finally, C computes $\sigma_{i}=V_{i}^{x_{i}}\cdot H_{1}{\left(ID_{i}\right)}^{s}$ and returns a signature *σ*_*i*_ on *m*_*i*_ to $A_{2}$.
- *Forgery*: $A_{2}$ eventually produces a valid forgery $\left({\left\{m_{i}^{*},ID_{i}^{*},pk_{i}^{*},\sigma_{i}^{*}\right\}}_{i=1}^{n},\sigma *\right)$. Suppose that the individual signature $\sigma_{t}^{*}$ is not returned by *Sign-query* on identity $ID_{t}^{*}$ and message $m_{t}^{*}$, where *t* ∈ {1, …, *n*}. If $ID*\neq ID_{t}^{*}$ or $V*=H_{3}\left(m_{t}^{*},ID*,pk*\right)$ is not found in $L_{3}$, C aborts. Otherwise, C looks up the lists $L_{1}$ and $L_{3}$ to obtain the tuples (ID*, y*, *H*_1_(ID*)) and $\left(m_{t}^{*},ID*,pk*,z*,g^{bz*}\right)$, respectively. Since $\sigma_{t}^{*}$ is valid, the following equation must hold:
  $$\begin{array}{ll} \sigma_{t}^{*} & ={\left(V*\right)}^{a}H_{1}{\left(ID*\right)}^{s}={\left(g^{bz*}\right)}^{a}H_{1}{\left(ID*\right)}^{s}\\ & ={\left(g^{ab}\right)}^{z*}H_{1}{\left(ID*\right)}^{s}. \end{array}$$
  Thus, C can use the master key *msk* = *s* to calculate the CDH value
  $$g^{ab}={\left(\frac{\sigma_{t}^{*}}{H_{1}{\left(ID*\right)}^{s}}\right)}^{{\left(z*\right)}^{-1}}.$$

Here, we also analyse the probability of C using $A_{2}$’s forgery to successfully solve the CDH instance. C completes the entire simulation if the following events occur.

- ID* has never appeared during secret value queries. The probability is at least ${\left(1-\frac{1}{q_{3}}\right)}^{q_{usk}}$.
- ID* has never appeared during signing queries. The probability is at least ${\left(1-\frac{1}{q_{3}}\right)}^{q_{s}}$.
- In the forgery phase, at least one of *n* identities $\left\{ID_{1}^{*},\ldots ,ID_{n}^{*}\right\}$ is the target user’s identity ID*. The probability of $ID*=ID_{t}^{*}$, where *t* ∈ {1, …, *n*}, is at least $1-{\left(1-\frac{1}{q_{3}}\right)}^{n}$.
- V* can be found in $L_{3}$. The probability is at least $\left(1-\frac{q_{3}}{p}\right)$.

Therefore, the overall success probability of C solving the CDH problem is

$$\begin{array}{ll} \varepsilon_{2}^{{}^{\prime }} & \geq {\left(1-\frac{1}{q_{3}}\right)}^{q_{usk}}{\left(1-\frac{1}{q_{3}}\right)}^{q_{s}}\left(1-{\left(1-\frac{1}{q_{3}}\right)}^{n}\right)\left(1-\frac{q_{3}}{p}\right)\varepsilon_{2}\\ & ={\left(1-\frac{1}{q_{3}}\right)}^{q_{usk}+q_{s}}\left(1-{\left(1-\frac{1}{q_{3}}\right)}^{n}\right)\left(1-\frac{q_{3}}{p}\right)\varepsilon_{2}. \end{array}$$

Since the CDH problem is intractable, the success probability of $A_{1}$ or $A_{2}$ attacking our CLAS scheme is negligible. According to Theorem 1 and Theorem 2, the following theorem is easily obtained.

**Theorem 3**. If the CDH assumption holds, then our improved CLAS scheme satisfies existential unforgeability in the random oracle model.

**Theorem 4**. Our CLAS scheme is secure under coalition attacks.

**Proof**. If an aggregate signature *σ* is valid, then the following equation must hold:

$$\begin{array}{ll} \sigma & =H\left(e\left(V_{1},pk_{1}\right)e\left(H_{1}\left(ID_{1}\right),P_{pub}\right),\ldots ,e\left(V_{n},pk_{n}\right)e\left(H_{1}\left(ID_{n}\right),P_{pub}\right)\right)\\ & =H\left(e\left(\sigma_{1},g\right),\ldots ,e\left(\sigma_{n},g\right)\right). \end{array}$$

Since the hash function *H* satisfies collision resistance, we conclude that

$$e\left(\sigma_{i},g\right)=e\left(V_{i},pk_{i}\right)e\left(H_{1}\left(ID_{i}\right),P_{pub}\right),\;i=1,\ldots ,n.$$

It shows that each individual signature *σ*_*i*_ participating in the aggregation is valid.

Meanwhile, if every individual signature *σ*_*i*_ is valid, the following equations hold:

$$e\left(\sigma_{i},g\right)=e\left(V_{i},pk_{i}\right)e\left(H_{1}\left(ID_{i}\right),P_{pub}\right),\;i=1,\ldots ,n.$$

Hence, we have

$$\begin{array}{ll} \sigma & =H\left(e\left(\sigma_{1},g\right),\ldots ,e\left(\sigma_{n},g\right)\right)\\ & =H\left(e\left(V_{1},pk_{1}\right)e\left(H_{1}\left(ID_{1}\right),P_{pub}\right),\ldots ,e\left(V_{n},pk_{n}\right)e\left(H_{1}\left(ID_{n}\right),P_{pub}\right)\right). \end{array}$$

This shows that the resulting aggregate signature *σ* generated by *σ*_*i*_(*i* = 1, …, *n*) is also valid.

The above analysis indicates that if the internal signers exchange each other’s individual signature value during aggregate signature generation, the collision resistance of *H* ensures that the final aggregate signature could not pass the signature verification equation. In other words, an aggregate signature is valid in our CLAS scheme if and only if each individual signature used to generate the aggregate signature is also valid. Hence, our CLAS scheme can resist coalition attacks.

### 4.4 Performance analysis

We compare our CLAS scheme with other similar schemes [27, 30, 31, 35, 39, 42–46] in terms of performance and security properties. In Table 1, we give the description of some symbols that need to be used in this subsection.

**Table 1:** Definition of some notations.

Symbol	Definition
*Size*	the overall length of an aggregate signature
*Sign*	the computational cost of the algorithm **Sign**
*AggVerify*	the computational cost of the algorithm **AggVerify**
*Coalition attack*	Can a scheme resist coalition attacks?
*n*	the overall number of signers participating in the aggregation
|*G*_1_|	the length of an element in *G*_1_
*P*	the computational cost of a bilinear pairing operation
*E*	the computational cost of an exponentiation calculation

#### 4.4.1 Performance comparison

Because the computational costs of multiplication, hash function and other cryptographic operations are relatively small, we focus on the time-consuming bilinear pairing and exponentiation operations when analysing the performance. Table 2 shows a detailed performance comparison between our CLAS scheme and the related CLAS schemes, where the required symbols are given in Table 1.

**Table 2:** Performance comparison of some CLAS schemes.

Scheme	Sign	AggVerify	Size	Coalition attack
Scheme of Cheng et al. [44]	3*E*	3*P*+2*nE*	(*n* + 1)|*G*_1_|	No
Scheme of Zhang et al. [43]	2*E*	2n*P*+2*nE*	|*G*_1_|	Yes
Scheme of Liu et al. [27]	3*E*	3*P*+2*nE*	2|*G*_1_|	No
Scheme of Xiong et al. [42]	3*E*	3*P*+2*nE*	(*n* + 1)|*G*_1_|	No
Scheme of He et al. [45]	3*E*	3*P*+2*nE*	(*n* + 1)|*G*_1_|	No
Scheme of Du et al. [30]	4*E*	4*P*+2*nE*	2|*G*_1_|	No
Scheme of Chen et al. [31]	4*E*	4*P*+2*nE*	(*n* + 1)|*G*_1_|	No
Scheme of Chen et al. [35]	4*E*	4*P*+2*nE*	2|*G*_1_|	No
Scheme of Kumar et al. [39]	4*E*	3*P*+2*nE*	(*n* + 1)|*G*_1_|	No
Scheme of Li et al. [46]	4*E*	3*P*+2*nE*	(*n* + 1)|*G*_1_|	No
Our scheme	*E*	*nP*	|*G*_1_|	Yes

From Table 2, for the computational cost of an individual signature generation, our CLAS scheme needs to perform only one exponentiation operation. Zhang et al.’s scheme [43] needs to perform 2 exponentiations. Cheng et al.’s scheme [44] requires 3 exponentiations, as do the schemes of Liu et al. [27], Xiong et al. [42] and He et al. [45]. The other five CLAS schemes [30, 31, 35, 39, 46] need to perform 4 exponentiations.

In our CLAS scheme, the verification equation for an aggregate signature

$$\begin{array}{c} \sigma =H(e\left(V_{1},pk_{1}\right)e\left(H_{1}\left(ID_{1}\right),P_{pub}\right),\ldots ,e\left(V_{n},pk_{n}\right)e\left(H_{1}\left(ID_{n}\right),P_{pub}\right)), \end{array}$$

where *e*(*H*_1_(*ID*_*i*_), *P*_*pub*_)(*i* = 1, …, *n*), can be pre-computed. During the aggregate signature verification, our CLAS scheme requires *n* bilinear pairings. The scheme of Chen et al. [35] requires 4 bilinear pairings and 2*n* exponentiations, as do Du et al.’s scheme [30] and Chen et al.’s scheme [31]. The CLAS scheme in [27, 39, 42, 44–46] requires 3 bilinear pairings and 2*n* exponentiations, while Zhang et al.’s scheme [43] requires 2*n* bilinear pairings and 2*n* exponentiations.

An aggregate signature is only one element of *G*_1_ in our CLAS scheme and Zhang et al.’s scheme [43]. In Liu et al.’s CLAS scheme [27], an aggregate signature is 2 elements in *G*_1_, which is the same as in Du et al.’s scheme [30] and Chen et al.’s scheme [35]. In the other six CLAS scenarios [31, 39, 42, 44–46], an aggregate signature consists of (*n* + 1) elements in *G*_1_.

Except for our CLAS scheme and Zhang et al.’s scheme [43], all other CLAS schemes [27, 30, 31, 35, 39, 42, 44–46] are insecure against coalition attacks. However, our CLAS scheme is superior to Zhang et al.’s scheme [43] in terms of computational performance.

#### 4.4.2 Experimental evaluation

**Fig 1:**
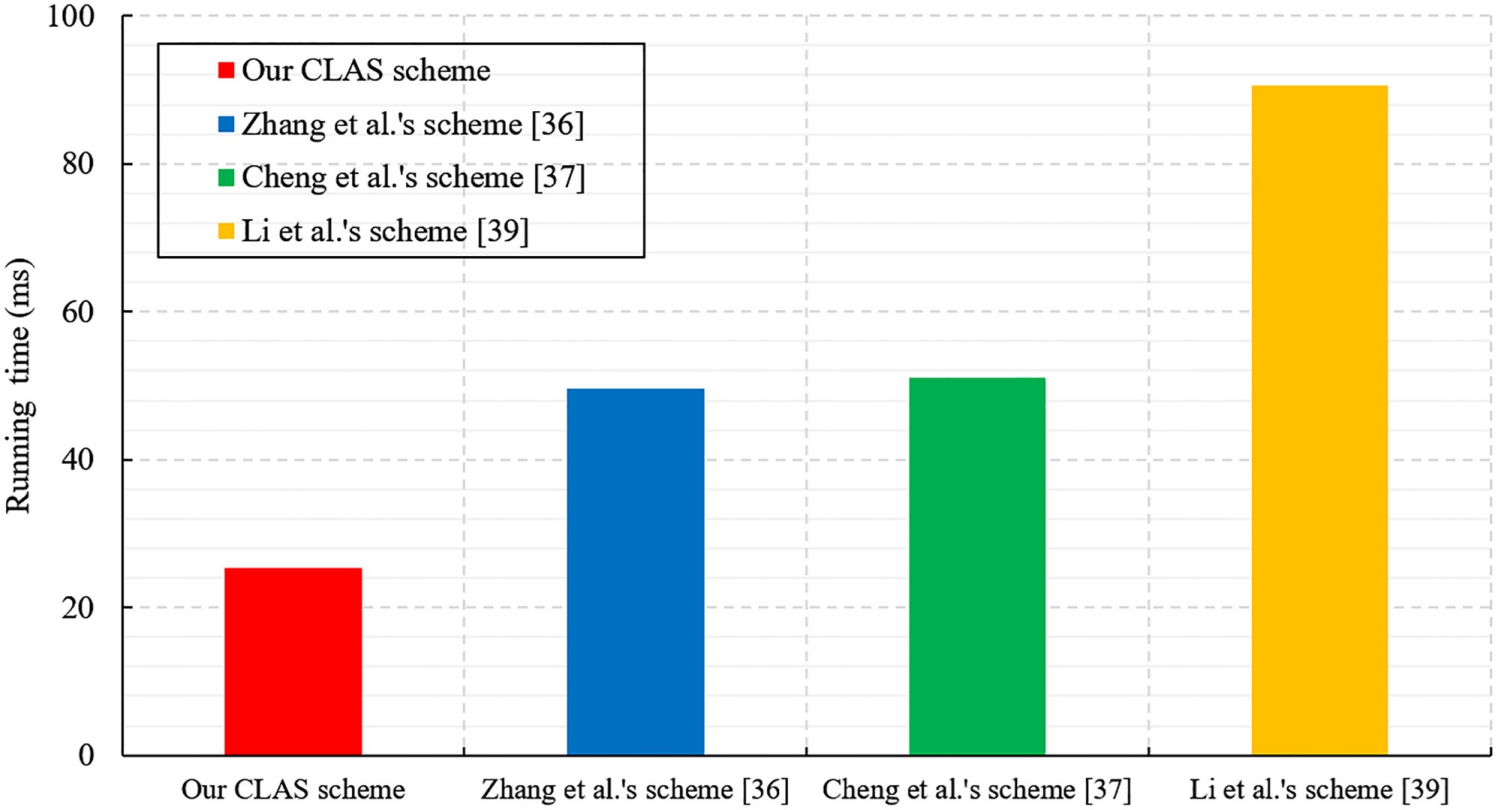
Time cost of individual signature generation.

**Fig 2:**
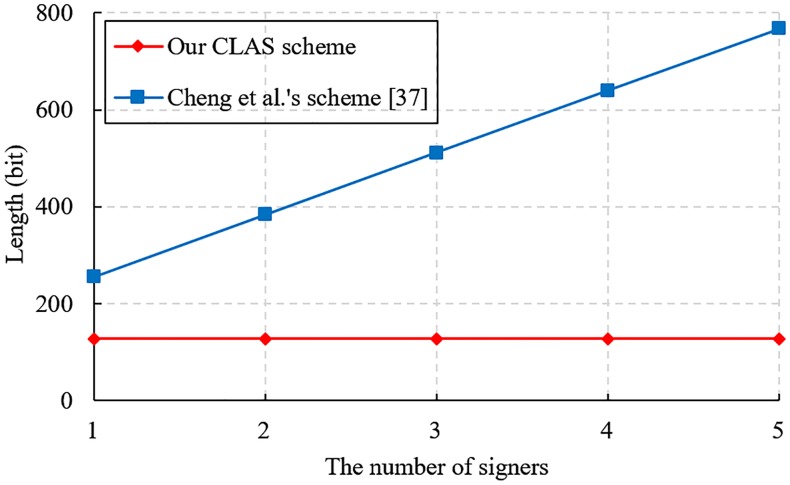
Comparison of communication cost.

**Fig 3:**
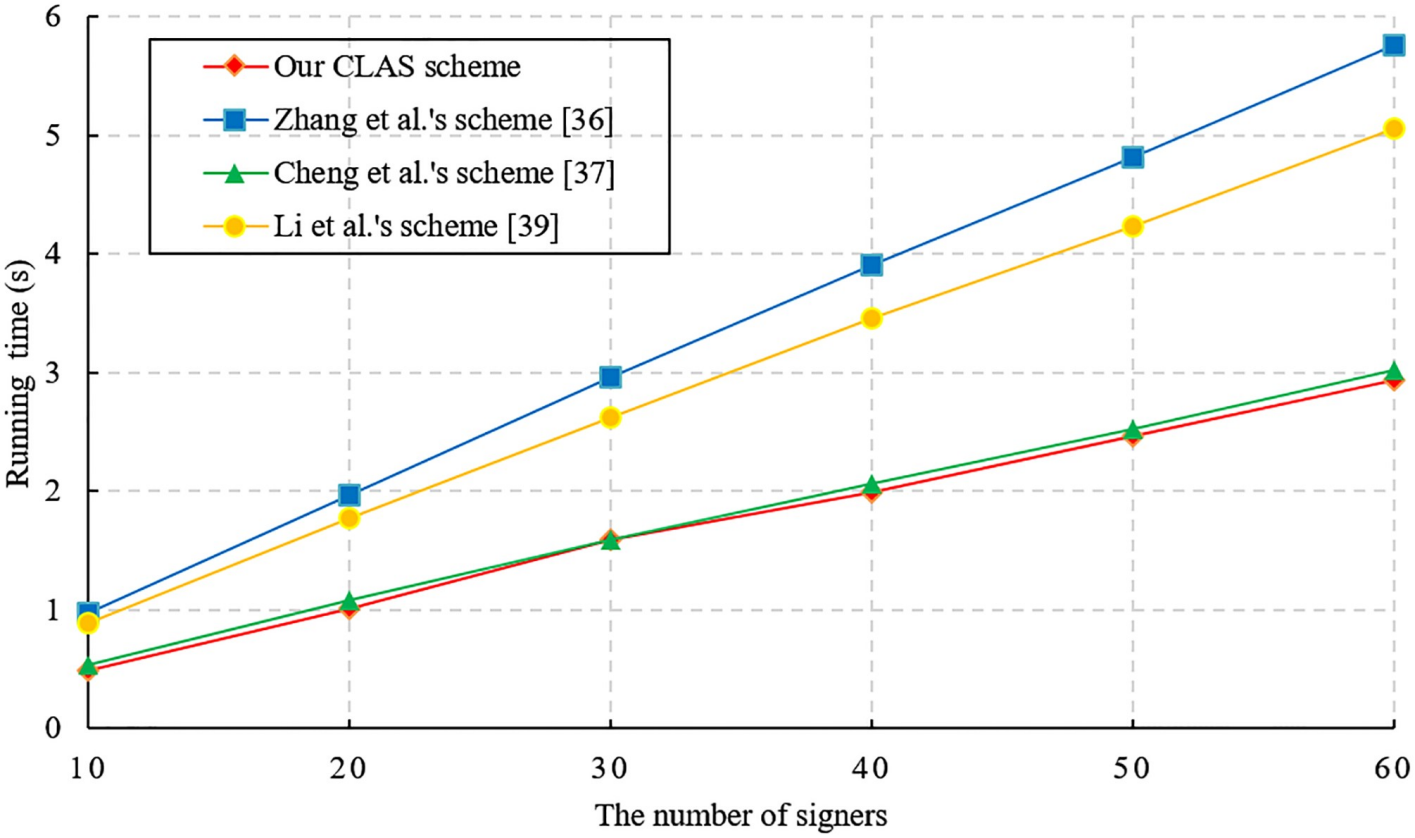
Time cost of aggregate signature verification.

We also conduct performance evaluation experiments on the proposed CLAS scheme and other CLAS schemes [43, 44, 46]. The simulated environment is set up on a laptop with the Windows 10 operating system, and the main configuration is Intel(R) Core(TM) i7-6500 CPU @ 2.59 GHz and 8 GB RAM. To execute the bilinear pairing operation quickly, we choose the curve *a.param* of type A in the PBC-0.47-VC library. The detailed simulation results are shown in Figs 1, 2 and 3.

Fig 1 shows that each signer needs approximately 26 ms to generate an individual signature in our CLAS scheme. The scheme of Zhang et al. [43] needs approximately 49 ms. Cheng et al.’s scheme [44] requires approximately 52 ms, and Li et al.’s scheme [46] requires approximately 91 ms. Therefore, our CLAS scheme requires the least computational cost when generating an individual signature.

The communication overhead of a CLAS scheme mainly relies on the size of the aggregate signature. Table 2 shows that our CLAS scheme and Zhang et al.’s scheme [43] have the same aggregate signature length, and Cheng et al.’s scheme [44] and Li et al.’s scheme [46] also have the same aggregate signature length. Fig 2 shows that the size of an aggregate signature in our CLAS scheme is fixed at 128 bits, while the length of an aggregate signature in Cheng et al.’s scheme [44] increases linearly with the number of signers. Hence, our CLAS scheme greatly reduces the communication cost.

Fig 3 shows that the computational cost of verifying an aggregated signature is linearly increasing with the total number of signatories. Notably, the verification cost of an aggregate signature is less than the total cost of *n* individual signature verifications participating in the aggregation. Compared with other CLAS schemes [43, 44, 46], Fig 3 shows that our CLAS scheme has lower computational overhead when verifying aggregate signatures.

In summary, the results of the performance evaluations performed in the above experiments are consistent with the theoretical analysis results in Table 2. From the above analysis, we can see that our improved CLAS scheme has lower computational overhead, shorter signature length and higher security.

## 5 Conclusions

Cheng et al. [44] proposed an efficient CLAS scheme and proved its security. In this paper, we demonstrate that their CLAS scheme [44] cannot resist coalition attacks from internal signers. To prevent these attacks, we design an enhanced CLAS scheme that is secure under coalition attacks. Under the CDH assumption, our improved scheme is proved to satisfy existential unforgeability in the random oracle model. In addition, our CLAS scheme can aggregate *n* individual signatures into a short signature which is only an element in *G*_1_. More importantly, the length of an aggregate signature is fixed and has nothing to do with the total number of signers participating in the aggregation. Therefore, our CLAS scheme is very suitable for network environments with limited resources, such as the Internet of vehicles, and wireless communication networks [50–52].

## Supporting information

S1 Data Time cost of individual signature generation. (XLSX)

S2 Data Comparison of communication cost. (XLSX)

S3 Data Time cost of aggregate signature verification. (XLSX)
